# Functional characterization of the lysosomal membrane protein TMEM192 in mice

**DOI:** 10.18632/oncotarget.17514

**Published:** 2017-04-28

**Authors:** Thuy Linh Nguyen, Janna Schneppenheim, Sönke Rudnik, Renate Lüllmann-Rauch, Christian Bernreuther, Irm Hermans-Borgmeyer, Markus Glatzel, Paul Saftig, Bernd Schröder

**Affiliations:** ^1^ Biochemical Institute, Christian Albrechts University of Kiel, Kiel, Germany; ^2^ Institute of Anatomy, Christian Albrechts University of Kiel, Kiel, Germany; ^3^ Institute of Neuropathology, University Medical Center Hamburg-Eppendorf, Hamburg, Germany; ^4^ Center for Molecular Neurobiology, University Medical Center Hamburg-Eppendorf, Hamburg, Germany

**Keywords:** transmembrane protein 192, lysosome, lysosomal membrane, autophagy, proteolytic processing

## Abstract

The Transmembrane protein 192 (TMEM192) is a lysosomal/late endosomal protein initially discovered by organellar proteomics. TMEM192 exhibits four transmembrane segments with cytosolic N- and C-termini and forms homodimers. Devoid of significant homologies, the molecular function of TMEM192 is currently unknown. Upon TMEM192 knockdown in hepatoma cells, a dysregulation of autophagy and increased apoptosis were reported. Here, we aimed to define the physiological role of TMEM192 by analysing consequences of TMEM192 ablation in mice. Therefore, we compared the biochemical properties of murine TMEM192 to those of the human orthologue. We reveal lysosomal residence of murine TMEM192 and demonstrate ubiquitous tissue expression. In brain, TMEM192 expression was pronounced in the hippocampus but also present in the cortex and cerebellum, as analysed based on a lacZ reporter allele. Murine TMEM192 undergoes proteolytic processing in a tissue-specific manner. Thereby, a 17 kDa fragment is generated which was detected in most murine tissues except liver. TMEM192 processing occurs after lysosomal targeting by pH-dependent lysosomal proteases. *TMEM192*-/- murine embryonic fibroblasts (MEFs) exhibited a regular morphology of endo-/lysosomes and were capable of performing autophagy and lysosomal exocytosis. Histopathological, ultrastructural and biochemical analyses of all major tissues of *TMEM192*-/- mice demonstrated normal lysosomal functions without apparent lysosomal storage. Furthermore, the abundance of the major immune cells was comparable in *TMEM192*-/- and wild type mice. Based on this, we conclude that under basal conditions *in vivo* the loss of TMEM192 can be efficiently compensated by alternative pathways. Further studies will be required to decipher its molecular function.

## INTRODUCTION

Lysosomes are central catabolic organelles of eukaryotic cells degrading cell-intrinsic constituents delivered by autophagy as well as exogenous, endocytosed material [1]. Turnover of all types of biological macromolecules is achieved by more than 50 acidic hydrolases which are enclosed by a single phospholipid bilayer [2]. In addition to providing a physical barrier, the lysosomal membrane harbours the vacuolar H^+^ ATPase for acidification of the lumen and controls efflux of monomeric degradation products into the cytosol [3]. Though more than 20 solute transport systems were demonstrated based on functional studies long ago, the identification and matching of the responsible proteins is still pending in many cases [4, 5]. In contrast to the soluble lysosomal hydrolases the knowledge of the protein composition of the lysosomal membrane has remained fragmentary for much longer. This reflects that classical protein purification approaches have been difficult to apply to the analysis of hydrophobic membrane proteins. Over the recent years important insights have been gained from systematic proteomic studies specifically analysing this subcompartment. These have identified several functionally uncharacterized integral membrane proteins with tentative lysosomal residence [2, 6, 7]. One of these candidates was the Transmembrane protein 192, TMEM192, or FLJ38482, according to the previous nomenclature, which was initially reported in lysosomal membranes isolated from human placenta [6].

Subsequently, lysosomal localization of human TMEM192 upon overexpression and also under endogenous conditions was confirmed [8]. In contrast to the majority of known lysosomal membrane proteins, human TMEM192 was found to be devoid of N-glycans [8]. Two dileucine motifs of the DXXLL type within the cytosolic N-terminal domain can independently initiate lysosomal targeting of the protein [9]. Only upon simultaneous ablation of both motifs, lysosomal delivery of TMEM192 was found to be impaired [9]. Furthermore, human TMEM192 forms homodimers [8], presumably mediated by the C-terminal domain of the protein [9].

As listed in the NCBI HomoloGene database, TMEM192 has orthologues also in non-vertebrates like *D. melanogaster* and plants like *A. thaliana*. However, it does not exhibit any homologies to functionally characterized protein families. A previous study has reported that TMEM192 plays a role in supporting the growth of tumour cells [10]. This was based on the observation that siRNA-mediated knockdown of TMEM192 expression induced growth inhibition, autophagy and apoptosis in HepG2 cells [10]. In addition TMEM192 was reported to be involved in the upregulation of autophagy by all-trans retinoic acid [11]. This was explained by an interaction with the putative tumor suppressor Tazarotene-induced gene 1 (TIG1) which was observed in a yeast two-hybrid screen [11]. However, the molecular function of TMEM192 in these specific contexts and within the lysosomal membrane in general has remained elusive to date.

Here, we aimed to provide insights into the physiological function of TMEM192 using mice as model organism. We have performed a detailed biochemical characterization of the murine TMEM192 protein in comparison to its predescribed human orthologue. Furthermore, we report the generation and phenotypic analysis of TMEM192-deficient mice. We show lysosomal function was not detectably affected by the absence of TMEM192. Importantly, no alteration of basal autophagy was demonstrated. Altogether, this shows that *in vivo* the loss of TMEM192 can be compensated by alternative pathways.

## RESULTS

### Murine TMEM192 is a lysosomal protein

**Figure 1:**
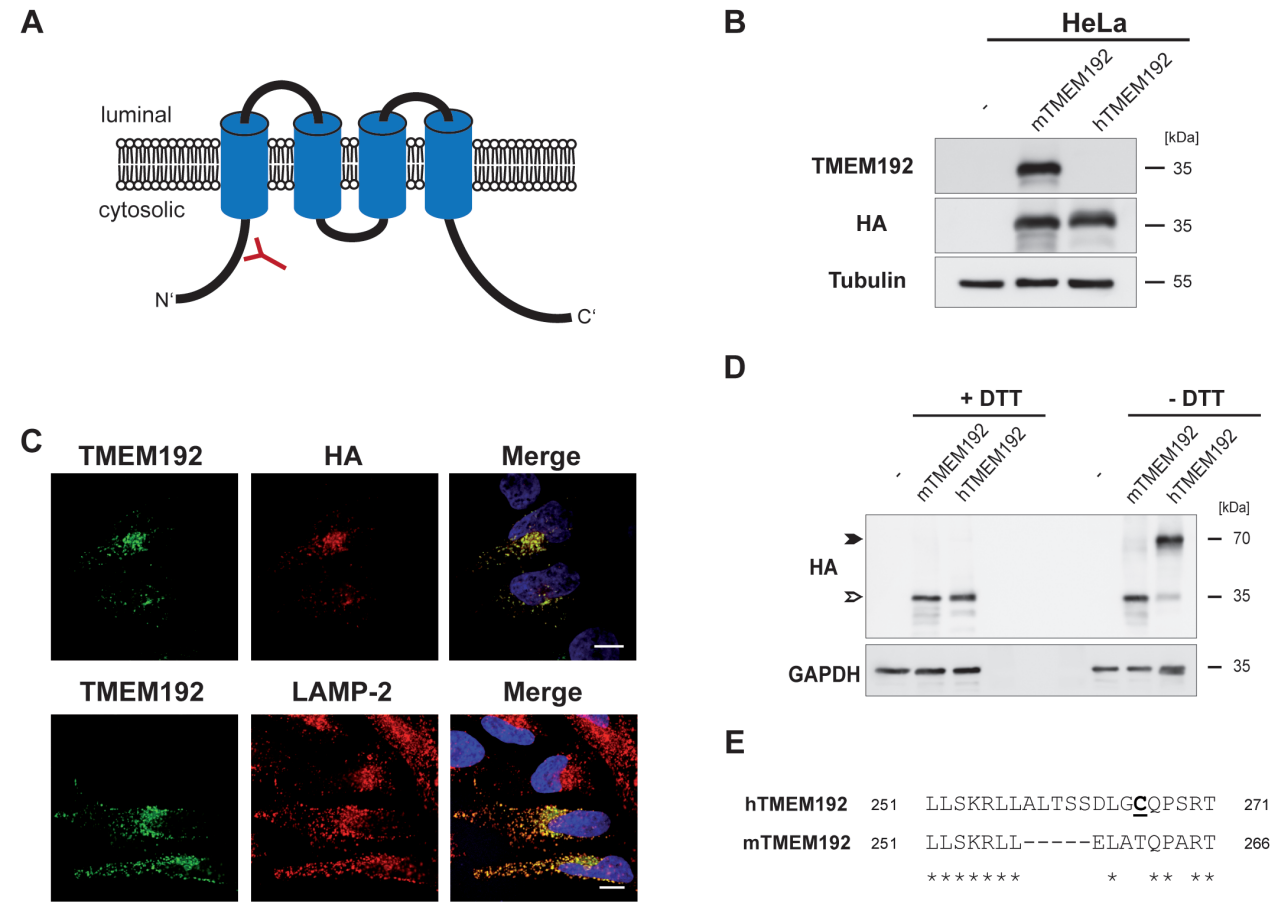
Biochemical properties of murine TMEM192 **A**. Schematic drawing of membrane topology and orientation of murine TMEM192. The position of the epitope used to generate polyclonal antisera for the detection of murine TMEM192 is indicated. **B**. Western blot analysis of HeLa cells transiently expressing human or murine TMEM192, both fused to an HA epitope, was performed in order to validate functionality and species specificity of the generated TMEM192 antibody. In order to confirm protein expression and equal protein loading the membrane was also stained with antibodies against the HA epitope and β-Tubulin. **C**. HeLa cells were transiently transfected with murine TMEM192 fused to an HA tag at its C-terminus. In order to validate the newly generated antibodies, the heterologously expressed murine TMEM192 was visualized by indirect immunofluorescence using the polyclonal TMEM192 antibody or the monoclonal antibody 3F10 binding to the HA epitope followed by appropriate fluorophore-conjugated secondary antibodies. As indicated, lysosomal localization was confirmed by detecting the lysosomal marker protein LAMP-2. Scale bar, 10 µm. **D**. To analyse disulphide-dependent dimerization of murine TMEM192, HeLa cells were transiently transfected with human or murine TMEM192. Aliquots of total lysates were denatured for 5 minutes at 95°C in the presence or absence of DTT and subjected to Western blotting. TMEM192 monomers and dimers were detected with monoclonal antibody 3F10 (HA) and are labelled with open and closed arrow-heads, respectively. Equal loading was confirmed by re-staining the membrane with anti-GAPDH. **E**.Alignment of the C-termini of human (aa251 - 271) and murine (aa 251 - 266) TMEM192. The cysteine residue C266 (bold, underline) previously identified to mediate dimerization of human TMEM is not present in the murine protein.

Prior to the generation and analysis of TMEM192-deficient mice, we aimed to confirm the applicability of this experimental system by comparing the biochemical properties of the murine TMEM192 orthologue with those of its human counterpart [8]. Comprising 271 and 266 amino acids, respectively, human and murine TMEM192 are identical to 78%. Equivalent to the human protein, the predicted topology of murine TMEM192 comprises four transmembrane segments with N- and C-termini facing the cytosol (Figure 1A). Since our available antibodies against human TMEM192 [8] did not recognize the murine protein, we generated a polyclonal antiserum against an N-terminal epitope of the murine orthologue (Figure 1A). This was validated by Western blot analysis of HeLa cells expressing murine TMEM192 (Figure 1B). We observed specific detection of overexpressed murine, but not human TMEM192 by this novel antibody. Under these overexpression conditions, the antibody also reliably visualized murine TMEM192 in situ by indirect immunofluorescence (Figure 1C). Thereby, we could confirm its residence in lysosomes based on its co-localisation with LAMP-2 (Figure 1C). Human TMEM192 forms homodimers that have been shown to be interconnected by disulphide bridges [8, 9]. Therefore, following an electrophoretic separation under non-reducing conditions mainly the dimer with an apparent molecular weight of 70 kDa (Figure 1D, closed arrow-head) was observed. In contrast, the murine protein was detected in its monomeric form also in the absence of the reducing agent dithiothreitol (DTT). This excludes the formation of disulphide bridges between different murine TMEM192 monomers. Since we had identified the C-terminal cysteine at position 266 (C^266^) to be part of the disulphide in human TMEM192, we aligned this part of the two proteins (Figure 1E). Interestingly, no cysteine residue is present in the C-terminus of murine TMEM192.

### Murine TMEM192 is ubiquitously expressed

**Figure 2:**
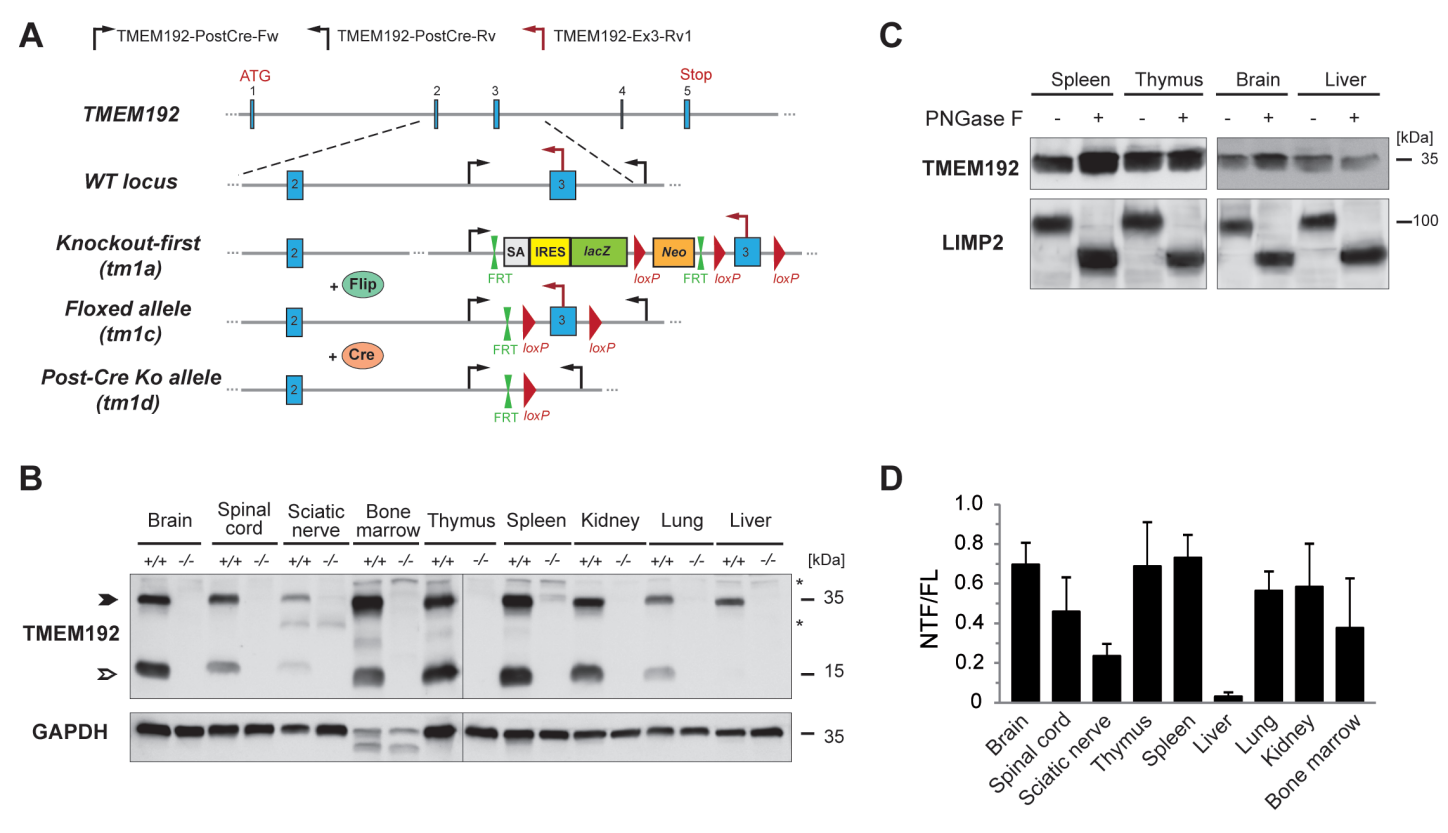
Murine TMEM192 is ubiquitously expressed and proteolytically processed **A**. Targeting strategy for disruption of the *TMEM192* gene. In the initially generated knockout-first allele (tm1a), expression of the *TMEM192* gene is disabled by a genetrap cassette which contains a β-Galactosidase reporter that is controlled by the endogenous TMEM192 promotor. This cassette was removed by breeding with Flip deleter mice leaving behind an allele with a loxP-flanked exon 3 (tm1c). Upon breeding with Cre deleter mice, this exon was excised generating a constitutive knockout allele (tm1d, *TMEM192*-/-). Positions of the three genotyping primers (TMEM192-PostCre-Fw, TMEM192-PostCre-Rv, TMEM192-Ex3-Rv1) that were used to distinguish mice with the tm1d allele and wild type mice are indicated in the scheme. **B**. Total organ lysates from wild type and *TMEM192*-/- mice were generated and analysed by Western blotting with antibodies against TMEM192. As a control for equal loading, the membrane was re-probed with anti-GAPDH. In addition to the monomeric TMEM192 full-length protein (FL, closed arrow-head), an N-terminal fragment (NTF, open arrowhead) derived from this protein was detected. *, unspecific band. **C**. N-glycosylation of endogenous murine TMEM192 was examined in total organ lysates from spleen, thymus, brain and liver from a wild type mouse. Prior to Western blotting, aliquots of lysates were incubated in absence or presence of PNGase F. Detection of TMEM192 was achieved with polyclonal TMEM192 antibody. As a control for glycosidase activity, deglycosylation of the lysosomal protein LIMP-2 was confirmed. **D**. Organ lysates of wild type mice were analysed by Western blotting for endogenous TMEM192 protein as in (B). Bands of the TMEM192 FL protein as well as of the NTF were quantified densitometrically and the ratio (NTF/FL) was calculated. Data are depicted as mean + SD and are based on the analysis of *n* = 5 mice.

Based on targeted ES cells obtained from the EUCOMM consortium, we generated TMEM192-deficient mice (Figure 2A). The initial tm1a allele was further modified by consecutive breeding with Flp- and Cre-deleter mice with ubiquitous, constitutive expression of these recombinases. Mice homozygous for the tm1d allele (*TMEM192*-/-), which exhibit a deletion of exon 3, were used for all further analyses aiming at characterizing the consequences of TMEM192 deficiency.

We performed Western blot analysis of equal protein amounts of different total organ lysates from wild type and *TMEM192*-/- mice to evaluate our antiserum for the detection of endogenous TMEM192 and to validate the knockout of TMEM192 at the protein level (Figure 2B). We observed a specific band with an apparent molecular weight of 35 kDa which was absent in all samples from *TMEM192*-/- mice (Figure 2B, closed arrowhead). The TMEM192 protein was detected in all murine tissues examined. Strongest expression of TMEM192 was found in bone marrow, thymus, spleen, kidney and brain. Weaker expression was seen in spinal cord, liver, lung and sciatic nerve (Figure 2B). According to bioinformatic analysis murine TMEM192 exhibits two NxS/T (N_76_PT, N_85_YT) consensus sites, of which only the second site is likely to be used for N-glycosylation due to the proline in the middle position of the first site [12]. However, PNGase F treatment of murine organ lysates also showed no change in apparent molecular weight of TMEM192 as seen before in human TMEM192 [6]. To verify proper activity of the added PNGase F enzyme the membrane was reprobed with antibodies against LIMP-2 showing a shift of the corresponding protein band (Figure 2C). Therefore, it can be concluded that murine TMEM192 is not modified with N-glycans.

As an unexpected finding, our TMEM192 antibody recognized an additional band with an apparent molecular weight of 17 kDa which was absent from the knockout tissues thereby confirming its specificity (Figure 2B, open arrowhead). In addition, a further, weak band at around 20 kDa was observed in bone marrow and thymus. Apart from liver, the 17 kDa TMEM192 band was prominently seen in all tissues analysed. Based on the recognition by our monospecific TMEM192 antibody, it can be concluded that the corresponding protein contains the N-terminal domain of TMEM192. No splice variants of this gene have been reported in the database, which could encode the observed 17 kDa protein species. Therefore, we hypothesized that this bands represent an N-terminal fragment (NTF) which is derived from the TMEM192 protein by proteolysis. We quantified the abundance of the TMEM192 fragment relative to the full-length protein in tissues obtained from *n* = 5 wild type mice (Figure 2D). This analysis demonstrated reproducibility of the extent of NTF generation in the different organs. In all analysed animals, only marginal amounts of the TMEM192 fragment were present in liver lysates. Furthermore, rather low levels were observed in peripheral (sciatic) nerves. The abundance in the other organs tested was in a comparable range with highest values detected in spleen, thymus and brain.

### Murine TMEM192 is proteolytically processed in lysosomes

**Figure 3:**
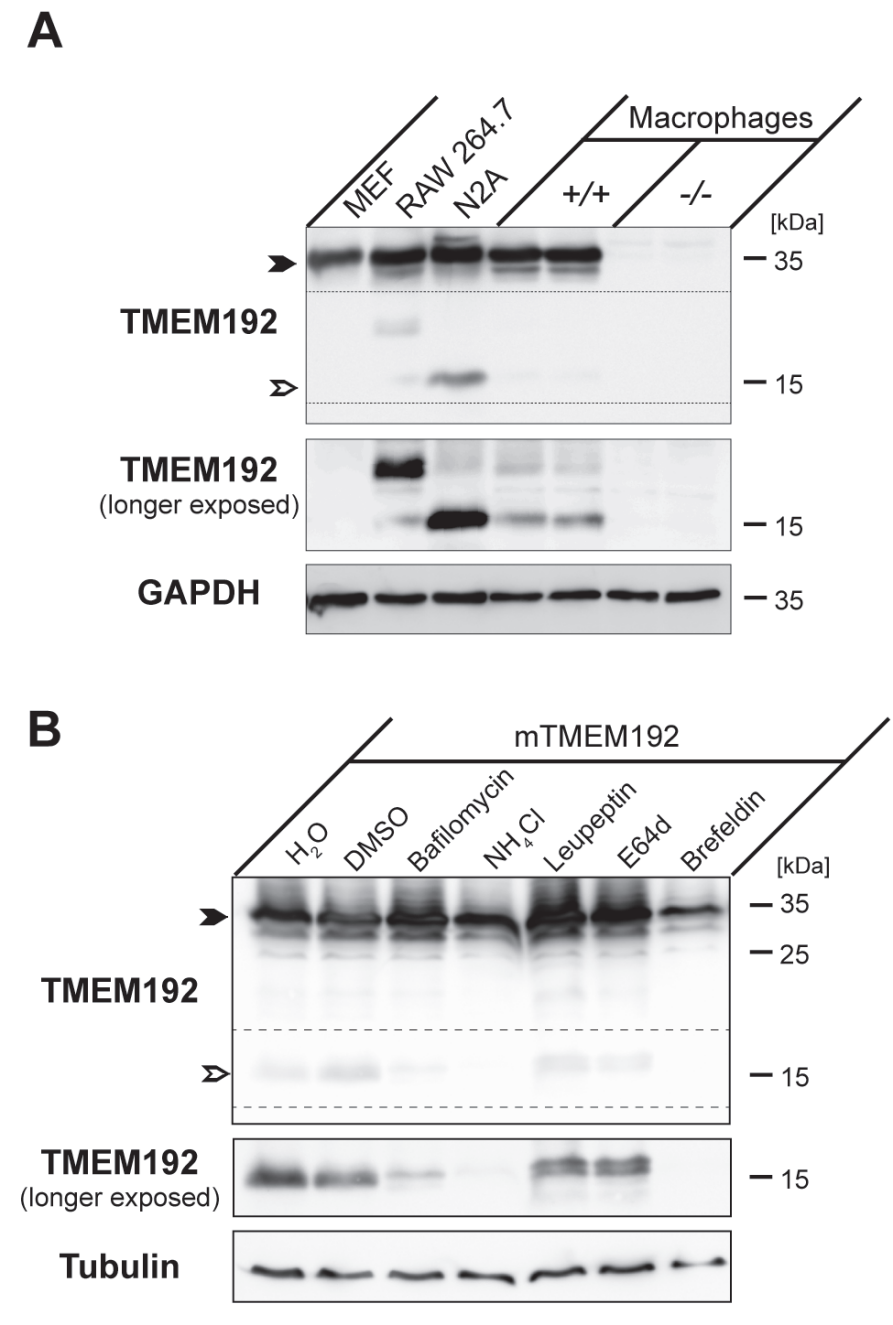
Proteolytic processing of murine TMEM192 occurs in lysosomes **A**. Abundance of the TMEM192 protein (closed arrow-head) and the derived TMEM192 N-terminal fragment (NTF, open arrow-head) was compared in murine embryonic fibroblasts (MEF), RAW 264.7 cells, N2A cells and primary Bone-marrow-derived macrophages from wild type mice (*+/+*). As a control for antibody specificity, macrophages from *TMEM192*-/- were included in the analysis. Total cell lysates of the depicted cell lines were subjected to Western blotting using the TMEM192 antibody. Equal loading was confirmed by re-probing the membrane with anti-GAPDH antibody. **B**. HeLa cells were transiently transfected with murine TMEM192. Cells were treated overnight with Bafilomycin (30 nM), NH_4_Cl (25 mM), Leupeptin (100 µM), E64d (40 µM), Brefeldin A (1 µg/ml) or H_2_O or DMSO as negative control. Aliquots of total cell lysates were analysed by Western blotting using the TMEM192 antibody. Due to a low processing efficiency of TMEM192 upon overexpression conditions, a longer exposure of the membrane was included to visualize the NTF and the effect of the different inhibitors on the generation of this fragment.

Based on the distinct tissue distribution, we also investigated presence of the TMEM192 NTF in different murine cell lines as well as primary bone marrow-derived macrophages (Figure 3A). In general, we found that in all cell types analysed the relative abundance of this protein fragment was much lower than in the tissue lysates. Whereas we failed to observe it in MEF cells, it was well detected in N2a neuroblastoma cells. Also primary macrophages exhibited small amounts of the NTF. Interestingly, in the immortalized macrophage cell line RAW 264.7 in addition to the weakly detected 17 kDa NTF an additional band at around 25 kDa was observed. This band pattern was reminiscent of that observed in Figure 2B upon analysis of murine bone marrow and thymus. Altogether, this indicates that proteolytic processing of TMEM192 can occur in a tissue and cell-type specific manner and that in some cases in addition to the major 17 kDa NTF additional processed forms can be observed.

In order to narrow down where in the cell TMEM192 is cleaved and which proteases are involved, we transiently transfected HeLa cells with murine TMEM192 and applied different inhibitors (Figure 3B). Similar to the rather low amounts of endogenous TMEM192 NTF present in the different murine cell lines, under these overexpression conditions only limited amounts of the proteolytic fragment could be detected. None of the applied protease inhibitors, targeting serine and cysteine (Figure 3B) as well aspartyl and metalloproteases (not shown) prevented generation of the NTF. Application of leupeptin and E64d slightly altered the electrophoretic migration of the NTF, but did not prevent its generation. However, interference with lysosomal acidification with Bafilomycin A1 or NH_4_Cl abolished TMEM192 processing. A similar effect was achieved by Brefeldin A which prevents post-ER trafficking and thus delivery to lysosomes of newly synthesized TMEM192. The effectiveness of the used compounds was confirmed in HeLa cells transiently expressing the invariant chain (CD74) of the MHCII complex (Supplementary Figure 1) which undergoes processing by endosomal/lysosomal proteases [13]. Altogether, these results demonstrate that proteolytic processing of TMEM192 occurs in lysosomes by proteases which are dependent on the acidification of this organelle. Since none of the individual protease inhibitors could block the generation of the NTF, presumably multiple proteases of different classes are involved in this process.

### Regular lysosomal functions in TMEM192-deficient fibroblasts

**Figure 4:**
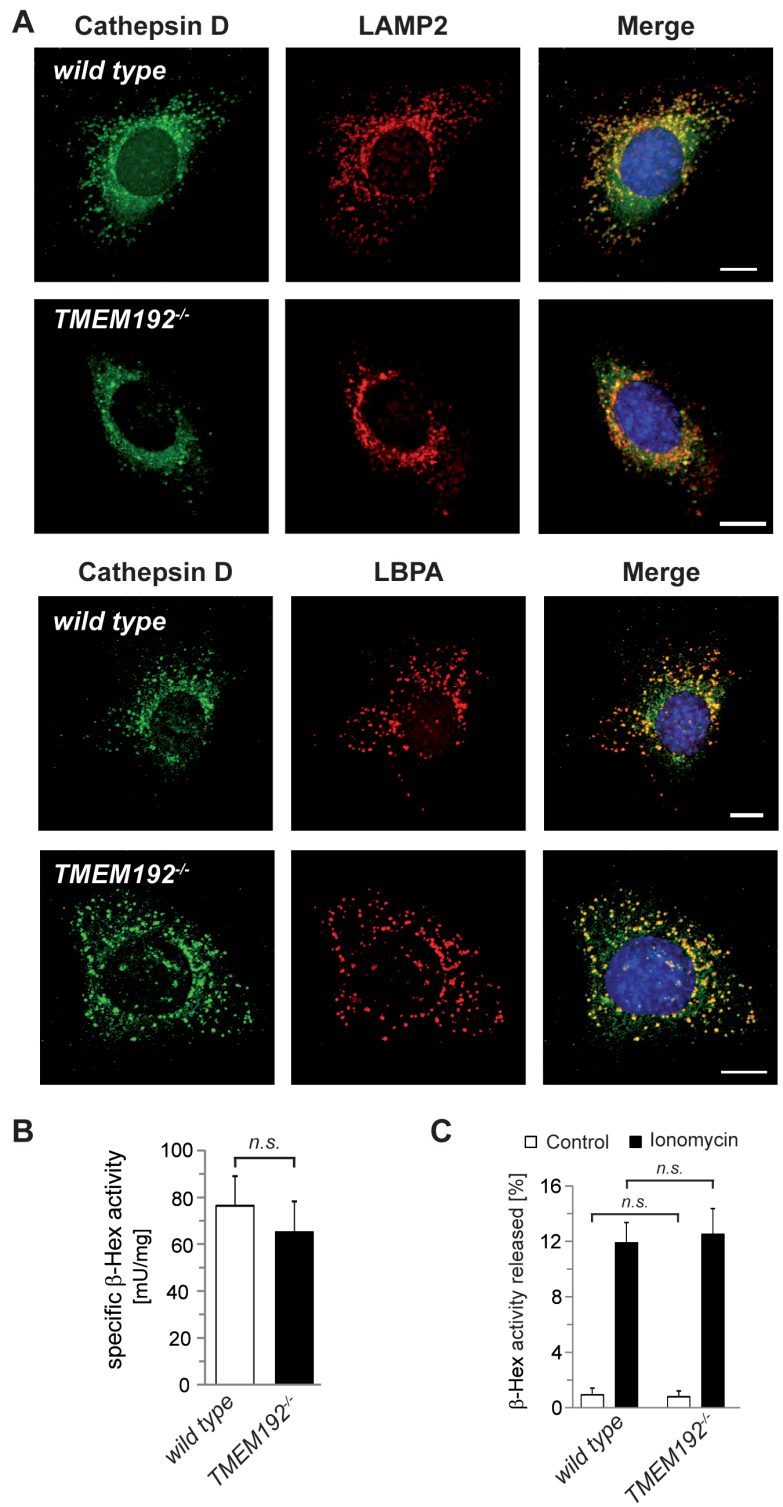
Loss of TMEM192 does not impair lysosomal function in TMEM192-deficient murine embryonic fibroblasts (MEFs) **A**. Wild type and *TMEM192*-/- MEFs were analysed for possible differences in lysosomal morphology by indirect immunofluorescence. Therefore, the luminal lysosomal protease Cathepsin D, the lysosomal membrane protein LAMP-2 and the late endosomal/lysosomal lipid LBPA were detected with specific antibodies and visualized with fluorochrome-conjugated secondary antibodies. Scale bar, 10 µm. **B**. The specific β-Hexosaminidase activity of total lysates from wild type and *TMEM192*-/- MEF cells was calculated based on the spectrophotometrically measured enzyme activity and the protein concentration. Results are based on *n* = 3 independent cell lines per genotype and are depicted as mean + SD. ns non-significant, unpaired, two tailed Student's t test. **C**. Wild type and *TMEM192*-/- MEF cells were treated with 10 µM ionomycine or an equivalent volume of DMSO in serum-free DMEM for 10 min at 37°C. Immediately after the incubation the medium was separated from the cells and cells were detached and lysed. The activity of β-Hexosaminidase was measured in the media as well as the cell lysates. The proportion (%) of released enzyme in relation to the total cellular enzyme pool (medium + lysate) was calculated for each sample and is depicted as mean + SD. *n* = 3; ns non-significant; unpaired, two tailed Student's t test.

To analyse the consequences of TMEM192 deficiency in single cells as well as *in vivo*, we used the generated *TMEM192*-/- mice. We performed a thorough cell biological analysis of *TMEM192*-/- MEF cells in comparison to wild type cells. To assess the morphology of the endo-lysosomal system, we visualised the intracellular distribution of the lysosomal protease Cathepsin D, the lysosomal transmembrane protein LAMP-2 and the late endosomal/lysosomal lipid LBPA (lysobisphosphatidic acid) by immunocytochemistry (Figure 4A). Size and intracellular distribution of lysosomes in the TMEM192-deficient cells resembled that in wild type MEFs. As a sensitive marker of lysosomal storage, we determined the specific activity of the lysosomal enzyme β-hexosaminidase activity in total cellular lysates of three independent cell lines of each genotype (Figure 4B). No difference was seen in the TMEM192 knockout MEFs, thereby excluding any relevant global lysosomal dysfunction in these cells.

**Figure 5:**
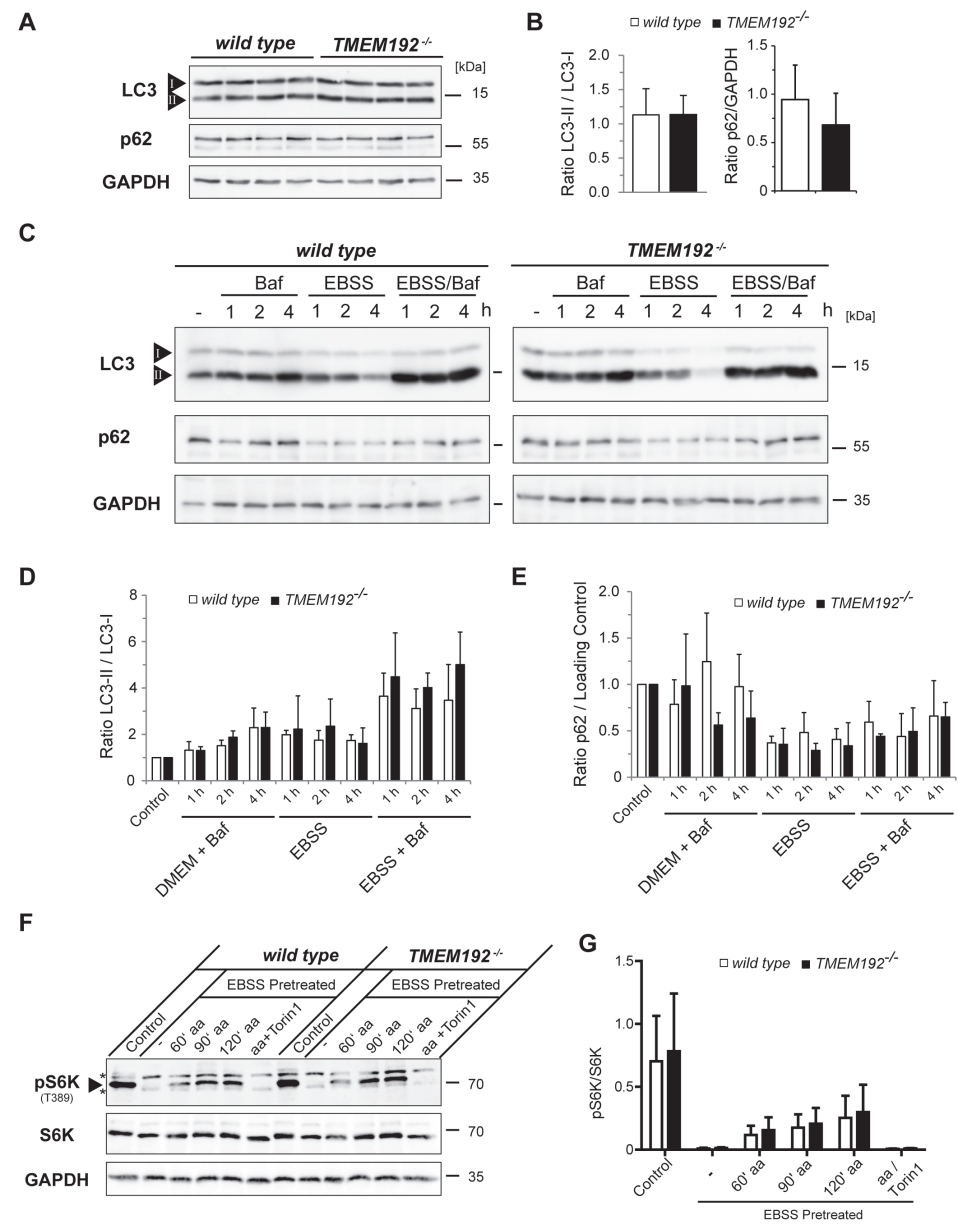
Comparable autophagic flux and regulation of mTOR activity in wild type and TMEM192-deficient murine embryonic fibroblasts (MEFs) **A**., **B**. Wild type and *TMEM192*-/- MEFs were analysed for basal steady-state levels of LC3-I and LC3-II and p62. Based on a densitometric quantification LC3-II/I and p62/GAPDH ratios were calculated and are depicted without any normalisation. Means + SD from *n* = 7-8 values per genotype are shown. **C**.-**E**. The capability to induce autophagy was compared in wild type and *TMEM192*-/- MEFs. Cells were starved in EBSS for 1, 2 and 4 h or kept in regular medium. To concomitantly inhibit lysosomal proteolysis, bafilomycin A1 (Baf) was added as indicated. Total cell lysates were subjected to Western blotting employing antibodies against LC3 and p62 as depicted from a representative experiment. To confirm equal loading the membrane was re-probed with GAPDH antibodies. A densitometric quantification of *n* = 3 experiments was performed as shown in (D) and (E). LC3-II/I ratios were determined and normalised to the value of the Control (regular medium, no Bafilomycin) of the respective genotype within each experiment (D). p62 values were corrected for putative differences in protein loading by calculating p62/GAPDH ratios. These values were also normalised to that of untreated cells as described above for the LC3-II/I ratios and are depicted in (E). All values are means + SD from *n* = 3 independent experiments. **F**., **G**. Intact regulation of mTORC1 activity in *TMEM192*-/- MEF cells. Wild type and *TMEM192*-/- MEFs were either left untreated (Control) or starved for one hour in EBSS (EBSS pretreated) in order to induce mTORC1 inactivation. Subsequently, mTORC1 reactivation was triggered by re-introducing amino acids (aa) via incubation in DMEM for 60, 90 and 120 min. Treatment with 250 nM Torin 1 during the reactivation period was used to silence mTORC1 kinase activity even in the presence of amino acids. The activity of mTORC1 was assessed based on the phophorylation status of its target p70S6 kinase (S6K) which was analysed by Western blotting as shown from a representative experiment in (F). The position of the band representing pS6K and two unspecific bands are marked with an arrowhead and asterisks, respectively. Densitometric quantification of phosphorylated S6K (pS6K) versus total S6K (pS6K/S6K) from *n* = 3 independent experiments is depicted in (G).

In addition to these analyses under steady-state conditions, we aimed to assess specific lysosomal functions. Therefore, we compared Ca^2+^-induced lysosomal exocytosis upon treatment of the cells with ionomycine (Figure 4C). The release of b-hexosaminidase was similar in wild type and *TMEM192*-/- cells indicating that the capability of lysosomes to fuse with the plasma membrane is not compromised by the loss of TMEM192. Furthermore, we analysed a potential impact on the initiation of autophagy and the degradation of autophagic cargo. Under basal conditions, LC3-II/I ratios were similar in wild type and TMEM192-deficient MEFs (Figure 5A, 5B). Furthermore, no accumulation of p62 as a potential indication of impaired autophagy was seen (Figure 5A, 5B). In order to compare the basal autophagic flux, cells were treated for 1, 2 and 4 h with Bafilomycin. In parallel, we starved the cells in EBSS for 1, 2 and 4 h in the absence or presence of Bafilomycin. Total cell lysates were analysed for LC3 conversion and levels of the autophagic cargo receptor p62 as depicted from a representative experiment in Figure 5C. A compilation of densitometric quantifications from *n* = 3 independent experiments is shown in Figure 5D, 5E. LC3-II/I and and p62/GAPDH ratios were normalised to the untreated control of the respective genotype within each experiment. Modulation of cellular p62 levels under these experimental conditions was not particularly pronounced and exhibited some interexperimental variability. However, the observed tendencies of a p62 reduction in starved cells were comparable in both genotypes. Importantly, the dynamics of LC3 conversion was very similar in wild type and TMEM192-deficient cells. Based on our analysis of three different time points, it can be concluded that the autophagic flux rate under basal as well as starvation conditions was not affected by the loss of TMEM192 in this cell type. In cells of both genotypes, the application of Bafilomycin led to a comparable stabilisation of LC3-II which indicates that autophagic cargo is subjected to lysosomal degradation. Therefore, we conclude that fusion of autophagosomes and lysosomes is not compromised upon TMEM192 deficiency. This was also reflected in a partial colocalisation of LC3 and LAMP-2 in cells starved for 3 h in the presence of Bafilomycin, which we observed to a similar degree in wild type and *TMEM192*-/- MEFs (Supplementary Figure 2).

We also analysed the impact of TMEM192-deficiency on activity of the autophagy regulating kinase mTORC1 (Figure 5F, 5G). Therefore, we assessed phosphorylation of the mTORC1 target p70 S6 kinase (S6K). Basal mTORC1 activity as well as its inactivation upon starvation and re-activation upon amino acid replenishment was comparable in wild type and *TMEM192*-/- cells. This demonstrates that lysosomal nutrient-sensing and the major regulatory pathway upstream of autophagy induction is intact in cells lacking TMEM192. Altogether we conclude that in MEF cells neither lysosomal functionality nor the regulation, initiation and conduction of autophagy are critically impaired in the absence of TMEM192.

### No obvious neurodegeneration and lysosomal pathology in the brain of TMEM192-deficient mice

**Figure 6:**
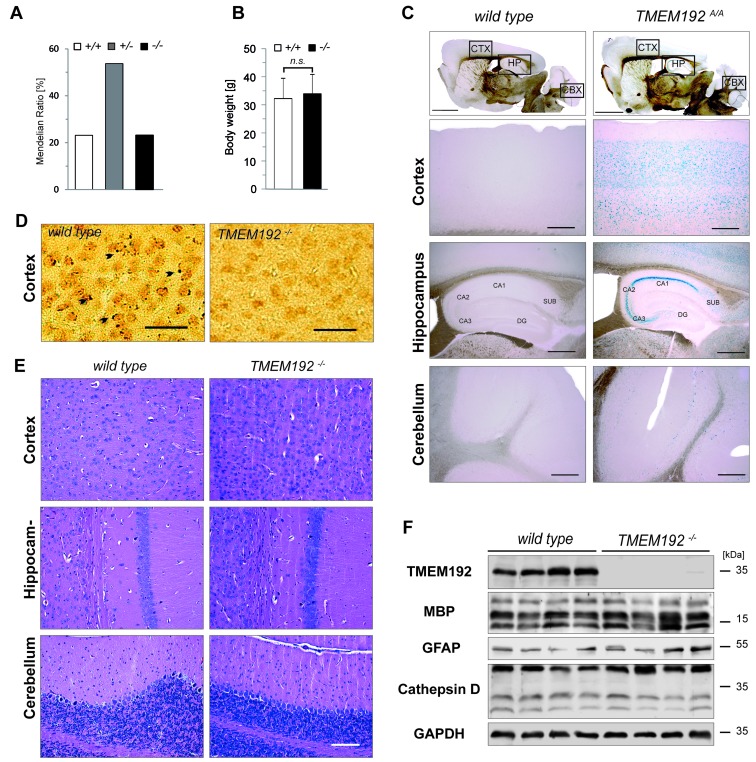
No obvious neurodegeneration and lysosomal pathology in the brain of TMEM192-deficient mice **A**. Genotype distribution among off-spring (*n* = 203 mice) from heterozygous matings of mice carrying the Post-Cre knockout allele (tm1d). Nomenclature is as follows: *+/+*, wild type; *+/-*, heterozygous; *-/-*, TMEM192 knockout. **B**. Comparison of body weight of age- and sex-matched wild type and *TMEM192*-/- mice. Mean + SD, n = 16 per genotype, ns non-significant, unpaired, two tailed Student's t test. **C**. Distribution of TMEM192 expression in mouse brain. Brain sections from homozygous mice with the tm1a allele (*TMEM192*A/A) and wild type mice as negative control were subjected to X-gal staining in order to visualize activity of the β-galactosidase reporter which is part of the tm1a allele. Mice were transcardially perfused with 4% PFA and cryosections were prepared. Scale bars: 2000 µm, overview; 500 µm,cortex (CTX) and cerebellum (CBX); 200 µm, hippocampus (HP). **D**. Immunohistochemical staining of TMEM192 in cortical brain sections from wild type mice. Cryosections from perfusion-fixed brains (4% PFA) were used. Diaminobenzidine was employed for visualization. To control for unspecific labelling, sections from *TMEM192*-/- brains were stained in parallel. TMEM192-positive vesicular structures are marked with arrow-heads. Scale bar = 250 µm. **E**. Representative brain sections from wild type and *TMEM192*-/- mice stained with hematoxylin and eosin demonstrated regular architecture of *TMEM192*-/- cortex, cerebellum and hippocampus (CA1 region). Staining was performed on paraffin sections from immersion-fixed brains (4% PFA). Scale bar: 100 µm. **F**. Total brain lysates from wild type and *TMEM192*-/- mice were generated and analysed by Western blotting with antibodies against TMEM192, myelin basic protein (MBP), glial fibrillary acidic protein, and cathepsin D. As loading control, the membrane was re-detected with anti-GAPDH. In **B**, **D, E** and **G** a two tailed Student's t test was performed. No statistical significant difference between wild type and TMEM192-deficient cells was observed.

In order to delineate the *in vivo* function of TMEM192, we performed a thorough phenotypic analysis of the *TMEM192*-/- mice. All phenotypic analyses were performed with mice carrying the post-cre tm1d allele which leads to a complete disruption of TMEM192 protein expression as demonstrated above (Figure 2B). Genotypes of mice born from heterozygous matings followed a Mendelian ratio (Figure 6A). Furthermore, the body weight of *TMEM192*-/- mice was similar to that of wild type controls (Figure 6B). We performed a broad histological analysis of all major tissues and organs. In many cases, this was complemented by examination at the ultrastructural level. This histopathological analysis initially performed in young adult (~ 15 weeks) mice was recapitulated in aged mice (>12 months). In the following figures, we have included data from brain, spleen and liver.

Having detected prominent expression of TMEM192 in brain, we tried to elucidate the distribution of this protein within this organ. Therefore, we utilised the lacZ reporter that is under the control of the TMEM192 promotor as part of the tm1a allele. Brain cryosections of mice homozygous for this allele were used for histochemical visualization of β-galactosidase activity using Xgal as substrate (Figure 6C). Labelling was most prominent in the hippocampus area. There, highest reporter activity was observed in the CA1 region, whereas very little was detected in the dentate gyrus (DG). Furthermore, lacZ-positive cells were observed in the cortex and within the Purkinje cell layer of the cerebellum (Figure 6C). Unfortunately, this approach was not applicable to other tissues than brain, since endogenous β-galactosidase activity caused significant background staining thereby preventing reliable discrimination of the lacZ activity. Therefore, we tried to apply our TMEM192 antibody, which reliably visualized overexpressed murine TMEM192 by indirect immunofluorescence (Figure 1C), for detection of endogenous TMEM192 in murine cell lines and tissues. In most cases, applicability of this antibody for *in situ* detection of endogenous TMEM192 was very limited. When used for immunohistochemical stainings of brain sections, it revealed a weak pattern of vesicular structures in wild type brain which was absent in TMEM192-deficient brain tissues. These vesicular structures resembled lysosomes and thereby support a lysosomal localisation of endogenous murine TMEM192 (Figure 6D).

We analysed if TMEM192 deficiency leads to histopathological changes in the brain, with a particular focus on the hippocampus, cortex and cerebellum where - based on the X-Gal staining - TMEM192 is expressed (Figure 6E). These areas showed no alteration in histoarchitecture in comparison to wild type, as seen in the depicted representative hematoxylin/eosin-stained sections. We also excluded gross defects in myelination, a major loss of neurons as well an astro- or microgliosis (Supplementary Figure 3). This was further substantiated by Western blot analysis demonstrating comparable levels of myelin basic protein (MBP) and glial fibrillary acidic protein (GFAP) in wild type and *TMEM192*-/- brains (Figure 6F). Since lysosomal storage at an early stage may histologically not be detected, we determined the specific β-Hexosaminidase activity in total brain lysates, which was similar in both genotypes (not shown). Furthermore, no accumulation or maturation defect of Cathepsin D was seen upon TMEM192 deficiency. Therefore, also biochemical analysis indicates regular lysosomal function in the central nervous system of *TMEM192*-/- mice.

### Regular liver and spleen architecture without signs of lysosomal dysfunction in TMEM192-/- mice

**Figure 7:**
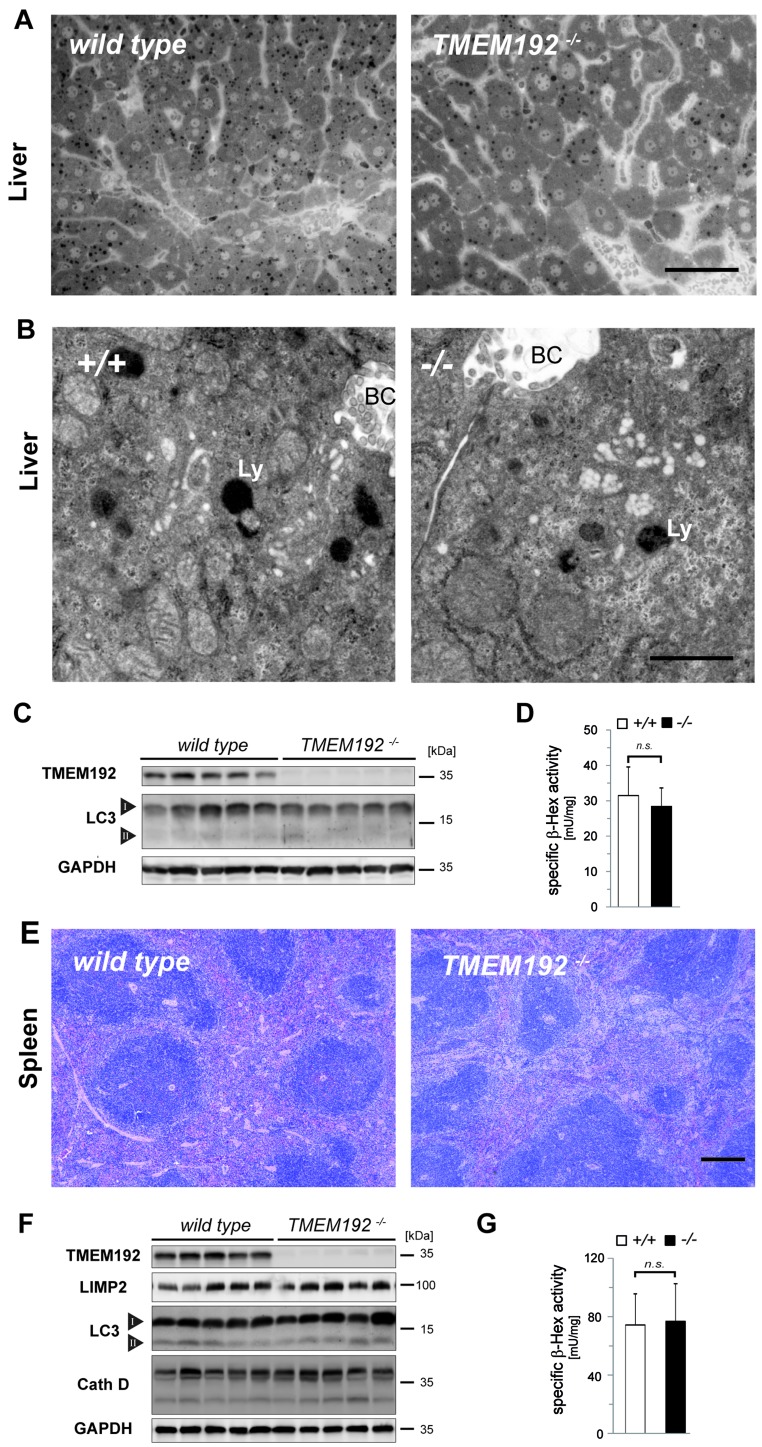
Regular liver and spleen architecture without signs of lysosomal dysfunction in TMEM192-deficient mice **A**. Representative histological sections from livers of wild type and *TMEM192*-/- mice. Tissue was fixed by transcardial perfusion with 3% glutaraldehyde. Semi-thin sections were prepared from araldite-embedded tissue and stained with toluidine blue. Scale bar, 50 µm. **B**. To analyse the ultrastructure of lysosomes in hepatocytes, transmission electron microscopy of ultrathin sections from perfusion-fixed (3% glutaraldehyde) and araldite-embedded wild type and *TMEM192*-/- livers was performed. BC, bile canaliculus; Ly, lysosome. Scale bar, 1 µm. **C**. To screen for differences in autophagy activation, total liver lysates of each genotype were analysed by Western blotting for LC3 conversion. The bands representing the two forms LC3-I and LC3-II are indicated. As a control for correct genotypes and protein loading, detection of TMEM192 and GAPDH was performed. **D**. The activity of the lysosomal enzyme β-Hexosaminidase was measured spectrophotometrically in total lysates of livers from wild type and *TMEM192*-/- mice. Means + SD of the specific enzyme activities are depicted, which were calculated for each sample based on the respective protein concentrations. Data are based on *n* = 6; unpaired, two tailed Student's t test, ns non-significant. **E**. Representative spleen sections from wild type and TMEM192-deficient mice. Paraffin sections from immersion-fixed (4% PFA) spleens were stained with hematoxylin and eosin. Scale bar, 250 µm. **F**. Western blot analysis of total spleen lysates from wild type and *TMEM192*-/- mice. The abundance of the lysosomal membrane protein LIMP-2, the autophagy marker LC3, and the lysosomal protease Cathepsin D was compared. In addition, TMEM192 and GAPDH were detected as controls. **G**. Specific activity of β-Hexosaminidase in total spleen lysates from wild type and *TMEM192*-/- mice. Data are based on *n* = 15; unpaired, two tailed Student's t test, ns non-significant.

A similar combination of morphological and biochemical approaches was applied to liver (Figure 7A-7D) and spleen (Figure 7E-7G) from TMEM192-deficient mice. Histological analysis of both organs revealed a regular histoarchitecture (Figures 7A and 7E). Electron microscopic examination of ultrathin sections from livers of wild type and *TMEM192*-/- mice revealed comparable morphology and size of lysosomes (Figure 7B). Furthermore, no accumulation of autophagosomes was observed. This was corroborated by Western blot analysis which did not detect an increase of LC3-II in TMEM192-deficient liver (Figure 7C) or spleen (Figure 7F). Based on this, we exclude a major alteration of baseline autophagy or an autophagosome-fusion defect. Similarly, specific β-Hexosaminidase activity was analysed in total lysates from liver (*n* = 6 per genotype, Figure 7D) and spleen (*n* = 15 per genotype, Figure 7G) and did not reveal significant differences between the two genotypes. Therefore, we conclude that TMEM192 deficiency in these tissues does not lead to any relevant lysosomal dysfunction.

Based on the prominent expression of TMEM192 in lymphatic tissues (Figure 2B), we quantified the major immune cell populations in the bone marrow, spleen, thymus and lymph node by flow cytometry (Table 1). We determined different stages of B and T lymphocytes differentiation in bone marrow, spleen and thymus, respectively. In addition, myeloid cell populations like macrophages, dendritic cells and granulocytes were quantified. Altogether, no significant differences between wild type and *TMEM192*-/- mice were observed demonstrating regular haematopoiesis and lymphocyte differentiation also in the absence of TMEM192. In conclusion, in the performed analyses we could not elucidate any relevant pathophysiological consequences of TMEM192 deficiency *in vivo*.

**Table 1:** Immune cell populations in lymphatic tissues of *TMEM192*-/- mice

Cell Type		*TMEM192*+/+		*TMEM192*-/-		*P*-value
		Mean	SD	Mean	SD	
		***Bone marrow*** (% of viable cells)				
**B cells**	B220^+^	16.8	2.0	16.0	4.5	0.731
**ProB/PreB**	B220^+^ IgM ^-^	6.7	1.3	6.8	2.3	0.915
**Immature B cells**	B220^+^ IgM ^+^	2.7	1.0	3.4	2.0	0.442
**Recirculating B cells**	B220^high^	7.4	1.3	5.8	1.4	0.073
		***Spleen***(% of viable cells)				
**B cells**	B220^+^	46.2	5.6	50.9	5.4	0.272
**T1 B cells**	CD21^low^ CD24^high^	6.6	2.8	6.6	1.7	0.988
**T2 B cells**	CD21^high^ CD24^high^	5.7	1.5	7.4	0.9	0.102
**mature B cells**	CD21^low^ CD24^low^	30.0	7.0	32.4	5.4	0.609
**T cells**	CD3^+^	29.5	5.7	24.4	4.3	0.198
**T helper cells**	CD3^+^ CD4^+^ CD8^-^	11.7	2.3	11.8	4.0	0.967
**T cytotoxic cells**	CD3^+^ CD4^-^ CD8^+^	17.3	3.5	11.9	4.0	0.087
**Dendritic cells**	CD11c^+^ MHCII^+^	5.1	1.3	6.3	1.1	0.203
**Granulocytes**	Gr1^+^	5.2	2.2	5.7	1.4	0.714
**Macrophages**	F4/80^+^	8.4	2.2	10.0	1.3	0.279
		***Spleen***(x10^6^splenocytes)				
**B cells**	B220^+^	37.2	13.7	43.4	7.5	0.5
**T1 B cells**	CD21^low^ CD24^high^	5.9	4.3	5.9	2.5	1.0
**T2 B cells**	CD21^high^ CD24^high^	5.0	2.5	6.5	2.0	0.4
**mature B cells**	CD21^low^ CD24^low^	23.2	6.6	27.3	2.6	0.3
**T cells**	CD3^+^	25.9	13.1	21.6	8.0	0.6
**T helper cells**	CD3^+^ CD4^+^ CD8^-^	10.1	4.8	10.0	3.0	1.0
**T cytotoxic cells**	CD3^+^ CD4^-^ CD8^+^	15.3	8.1	10.9	5.9	0.4
**Dendritic cells**	CD11c^+^ MHCII^+^	4.2	2.2	5.5	1.8	0.4
**Granulocytes**	Gr1^+^	4.6	2.8	5.0	2.1	0.9
**Macrophages**	F4/80^+^	7.5	3.8	8.8	3.0	0.6
		***Lymph nodes***(% of viable cells)				
**T cells**	CD3^+^	41.9	13.0	48.4	7.2	0.313
**T cytotoxic cells**	CD3^+^ CD4^-^ CD8^+^	18.3	5.8	22.5	4.9	0.203
**T helper cells**	CD3^+^ CD4^+^ CD8^-^	22.0	6.9	23.8	3.2	0.578
**B cells**	B220^+^	23.3	3.6	24.0	7.0	0.946
**Dendritic cells**	CD11c^+^ MHCII^+^	7.0	3.1	6.8	5.6	0.961
		Thymus(% of viable cells)				
**Double-positive T cells**	CD4^+^ CD8^+^	11.4	3.0	11.9	3.8	0.826
**CD4+ T cells**	CD4^+^ CD8^-^	82.5	5.1	80.3	6.4	0.613
**CD8+ T cells**	CD4^-^ CD8^+^	3.8	1.6	5.6	4.2	0.456
**Double-negative T cells**	CD4^-^ CD8^-^	2.4	1.6	2.2	1.3	0.908

Flow cytometric analysis of cells from n=4 (spleen, thymus) or *n* = 6 (bone marrow, lymph nodes) mice of each genotype was performed to determine the proportions of the indicated cell populations, which are shown as the percentage of viable cells (PI^-^) or absolute cell numbers. Results are presented as mean ± SD, unpaired two tailed Student's *t* test.

## DISCUSSION

The presented results indicate that most of the biochemical properties of human and murine TMEM192 show a significant similarity, though some differences were observed. Lysosomal residence could be confirmed for the murine protein. In a proteomic analysis of rat liver lysosomes also the rat orthologue was detected in this cellular compartment [7]. In agreement with a consistent lysosomal localization of the TMEM192 orthologues, the N-terminal of the two targeting motifs which we identified previously in the human protein [9] is also fully conserved in the murine and rat proteins. This is not the case for the second motif (DAQLL, human; DTQPL, mouse, rat) which may indicate that in rodents the first motif has a leading role. For the human protein, either of the two motifs was sufficient to facilitate lysosomal targeting of TMEM192 [9]. Interestingly, in contrast to the human protein [8, 9] no disulphide bridge formation between murine TMEM192 monomers was observed. However, we discussed previously whether this modification could also represent a post-lysis artefact which is facilitated by close proximity of the C-termini in a TMEM192 dimer since the corresponding residues are localised in the cytosolic part of the protein. The absence of the respective cysteine residue in murine and rat TMEM192 may argue for a limited functional relevance of the disulphide linkage, which should therefore be rather considered as a consequence and not a prerequisite of dimerization.

An unexpected finding was that murine TMEM192 is subjected to proteolytic processing after its delivery to lysosomes. Since this cleavage could be blocked in cells by different pretreatments in order to block lysosomal targeting or acidification, we exclude that this fragment is generated subsequent to cell lysis. Based on the size of the N-terminal cleavage product and the described role of lysosomal proteases, we assume that the cleavage site is localized within the first luminal loop of TMEM192. We assessed the contribution of several cathepsins by analysing tissue samples from mice deficient for different cathepsins (not shown). No impairment of TMEM192 processing was seen in these mice supporting the conclusion from the cell-based inhibitor experiments that multiple lysosomal proteases take part in this process. Proteolytic cleavage of human TMEM192 has not been reported so far which may have two reasons. First of all, studies on the human protein were for obvious reasons nearly exclusively based on cultured cell lines, where also in the case of the murine protein processing was much less prominent than in the tissue samples. Furthermore, the previously generated antibody detecting the human protein was raised against a C-terminal epitope [8]. Therefore, it seems likely that proteolysis of the human protein may have been overlooked in previous studies [8, 9] due to technical reasons.

Since the molecular function of TMEM192 is currently elusive, it can only be speculated how this is influenced by the proteolytic cleavage and if the processed form has a biological function. In general, it could also represent a degradation intermediate of TMEM192. However, in light of the significant steady-state levels of the NTF in relation to the full-length protein and its distinct molecular weight, this does not seem too likely. Interestingly, several examples of lysosomal polytopic membrane proteins that are distinctly processed by lysosomal proteases have been reported over the last years. These include the heparin-α-glucosaminide N-acetyltransferase (HSGNAT) [14], mucolipin-1 [15, 16], and the putative transport proteins DIRC2 [17] and CLN7 [18]. However, similar to TMEM192, in most cases the functional role of the cleavage events is not yet understood.

We have shown at the protein level that murine TMEM192 is ubiquitously expressed in mice. In a previous analysis by RT-PCR [10], liver was found to exhibit the highest TMEM192 transcript levels within a broad panel of organs. Interestingly, in our Western blot analysis murine liver contained comparably low amounts of TMEM192 protein with significantly higher levels in lymphatic tissues and brain.

In light of the ubiquitous presence of TMEM192, it may seem surprising that the loss of TMEM192 does not negatively affect general lysosomal functionality. No indications for a lysosomal storage, a dysregulation of autophagy or a failure to degrade autophagic cargo were observed in tissues of our *TMEM192*-/- mice or primary cells like MEFs or macrophages derived from these mice. Since a subtle compromise of lysosomal functions may become evident only after certain latency, our analysis also included aged mice (> 12 months). However, also in these animals no cellular phenotype or pathology associated with the TMEM192 deficiency was seen.

In contrast, Liu et al. had reported that siRNA-mediated knockdown of TMEM192 in HepG2 hepatoma cells induces autophagy and leads to growth inhibition [10]. This effect was not seen in the hepatic L02 cell line. Altogether, this could indicate that tumour cells are more susceptible to the loss of TMEM192 than normal cells. Possibly, certain compensatory proteins/pathways that have an overlapping molecular activity are missing or less abundant in tumour cells. Alternatively, tumour cells may have a higher demand of the specific function of TMEM192, possibly related to their rapid proliferation or due to the upregulation of specific pathways. In this context, the very recently reported link between TMEM192 and the retinoic-acid TIG1 may deserve further attention [11]. Autophagy and the supply of nutrients from lysosomal cargo degradation are known to play a critical role in cancer cells [19, 20]. With its four transmembrane segments and the ability to dimerise, a potential role in solute and/or nutrient translocation across the lysosomal membrane seems conceivable, which could be more critically required in rapidly dividing cells. In the study by Liu et al. experiments were mainly performed in one hepatoma cell line [10] strongly advocating further investigations in a broader panel of cell lines. In conclusion, additional studies will be required to decipher the molecular function of TMEM192 as well as its possible role in tumour cells.

## MATERIALS AND METHODS

### Antibodies and reagents

A monoclonal antibody against the HA (haemagglutinin) epitope tag (3F10) was purchased from Roche. Anti-GAPDH (glyceraldehydes-3-phosphate dehydrogenase) was obtained from Santa Cruz Biotechnology. Monoclonal antibodies against β-tubulin clone E7 and murine LAMP-2 (Abl93) were from Developmental Studies Hybridoma Bank (DSHB). The polyclonal antibodies against lysosomal marker LIMP-2 [21], human LAMP-2 (2D5) [22] and Cathepsin D (SII.10)[23]have been described previously. Anti-LBPA (6C4) was kindly provided by Jean Gruenberg (University of Geneva, Geneva, Switzerland). Anti-LC3 and anti-p62 were bought from MBL International Corporation and Enzo Life Sciences, respectively. Antibodies against GFAP and MBP were purchased from Sigma-Aldrich and Dako. A rat monoclonal antibody (In-1) against the N-terminus of murine CD74 was obtained from BD Biosciences. For the analysis of the mTORC1 activity, antibodies against phosphorylated (Thr-389) and total p70S6K (S6K) were from Cell Signaling Technology. Antisera against murine TMEM192 were produced by Pineda Antikörper-Service, Berlin. Rabbits were immunized with a synthetic peptide consisting of amino acids 21-42 of murine TMEM192 (DPLLDTQPLPHHSLQAQFRPRF). The obtained antisera were affinity-purified against the immobilized peptide. For immunoblotting, secondary antibodies coupled to horseradish peroxidase from Dianova were used. Fluorochrome-conjugated secondary antibodies (goat-anti-rabbit IgG coupled to Alexa 488, goat-anti-rat IgG and goat-anti-mouse IgG coupled to Alexa 594) were bought from Molecular Probes. To analyse the requirements for TMEM192 processing, the following compounds and inhibitors were employed: Bafilomycin A1 (Sigma), NH_4_Cl (Roth), Leupeptin (Sigma), E64d (Enzo), Pepstatin A (Sigma), Marimastat (Sigma), Brefeldin A (Enzo). Except for Leupeptin, E64d and NH_4_Cl which were dissolved in water, stock solutions from all other compounds were prepared in DMSO.

### Mice

Targeted ES cells (JM8A1.N3 cell line) were obtained from the EUCOMM consortium (Project ID 37139, Clone HEPD0785_6_G07) and provided by the Helmholtz Zentrum, Munich, Germany. Generation, breeding and analysis of mice was in line with local and national guidelines and has been approved by the local authorities (Amt für Verbraucherschutz, Lebensmittelsicherheit und Veterinärwesen, Hamburg, No. 59/13; Ministerium für Energiewende, Landwirtschaft, Umwelt und ländliche Räume, Kiel, V 312-72241.121-3). ES cells were injected into C57/BL6 blastocysts. The obtained chimeric mice were bred with C57/BL6 N Crl wild type mice to obtain germline transmission. The allele design is depicted in Figure 2A. In its original state (tm1a, Knockout-first), expression of TMEM192 is disrupted by a splice acceptor (SA) in the intronic sequence between exons 2 and 3. At the same time, a β-galactosidase reporter (lacZ) is expressed under the control of the endogenous TMEM192 promotor. Mice carrying this tm1a allele were exclusively used for immunohistochemical detection of lacZ activity in order to analyse the tissue distribution of TMEM192. Incomplete utilisation of a splice acceptor, as it is part of the genetrap cassette in the tm1a allele, can lead to minor amounts of residual protein expression. To avoid this, all phenotypic analyses reported in this study were performed on *TMEM192*-/- mice which exhibited the tm1d allele after Cre-mediated excision of exon 3. To obtain this allele, mice carrying the tm1a allele were consecutively bred with Flp- [24] and Cre-deleter mice [25] which constitutively express the respective recombinases under the control of a CMV promotor. Successful generation of the different intermediate alleles was confirmed by specific PCRs. Finally, the Flp and Cre transgenes were removed from founder mice with the tm1d allele by breeding. Animals for all phenotypic analyses were generated by heterozygous matings of mice with the TMEM192 tm1d allele. Thus, genotyping of the offspring aimed at differentiating the wild type and the tm1d knockout allele either in a homozygous or heterozygous state was performed. Therefore, a multiplex PCR amplifying specific 600 bp and 333 bp products from the wild type and the TMEM192 tm1d allele was conducted utilizing the following three primers: TMEM192-PostCre-Fw, 5‘-TGATGCTTTTGGCAAACAAATCTA-3‘; TMEM192-PostCre-Rv: 5‘-AGGAGTGCCAGCCTATAAGACACG-3‘; TMEM192-Ex3-Rv1: 5‘-GATTAGGATACAAACACGGCACA-3‘. In all experiments, either littermates or pairs of sex- and age-matched wild type and *TMEM192*-/- mice were compared.

### Plasmids

An expression constructs for human TMEM192 with a C-terminally fused HA epitope (hTMEM192-HA-pcDNA3.1/Hygro^+^) has been described previously [8]. The murine TMEM192 open reading frame was amplified from murine cDNA using the following primers: mTMEM192-HindIII-Fw, TGCCAAGCTTACGCCACCATG GCGGCGGCCGGCCGGCTGG; mTMEM192-HA-XhoI-Rv, GATCCTCGAGT GTCGTATGGGTAAGTCCTGGCTGGCTGAGTTGCC. The PCR product was inserted in pcDNA3.1/Hygro^+^ vector (Invitrogen) via the appended HindIII and XhoI restriction sites and the sequence of the final construct was verified (GATC Sequencing Service). Generation of an expression constructs of murine CD74 has been described before [26].

### Cell culture and transfection

HeLa cells (DSMZ) and Murine embryonic fibroblasts (MEFs) were cultivated in DMEM (Dulbecco's modified Eagle's medium, Gibco) supplemented with 10% (v/v) FCS (fetal calf serum, Biochrom), 100 units/ml penicillin (Sigma) and 100 µg/ml streptomycin (Sigma). MEFs were isolated from 13.5 day old *TMEM192*-/- and wild type embryos. Heads and internal organs were removed and the remaining embryonic tissues was minced and incubated in Trypsin-EDTA solution (Sigma) at 37°C for 15 min. Remaining tissue pieces were disintegrated by pipetting and transferred into prewarmed supplemented DMEM medium. After centrifugation and one step of washing, cells were plated in a 10 cm dish. Experiments were performed with primary MEF cells at an early passage (>10). Bone-marrow derived macrophages (BMDM) were differentiated from bone marrow cells obtained by flushing femurs and tibiae of wild type and *TMEM192*-/- mice. Cells were cultured in DMEM containing 20% (v/v) FCS, penicillin/streptomycin as described above and 50 ng/ml M-CSF (Immunotools). All cultivated cells were maintained in a humidified 5% CO_2_ /air atmosphere at 37°C.

HeLa cells were transiently transfected at semiconfluence using TurboFect (Thermo Fisher Scientific) corresponding to manufacturer's instructions. After 6-8 h incubation time, the transfection medium was replaced by fresh medium to reduce cytotoxicity. Cells were harvested or fixed 24 h post-transfection.

For the induction of autophagy in MEF cells, cells were washed twice with PBS before being starved in Earle's Balanced Salt Solution (EBSS, Gibco) for 1, 2 and 4 h. To prevent degradation of autophagocytosed cargo and reveal the autophagic flux, bafilomycin at a final concentration of 30 nM was added where indicated. After the incubation, the cells were immediately cooled down on ice and lysed as described below.

The regulation of mTORC1 activity was performed as reported previously [27]. In brief, cells were rinsed with phosphate-buffered saline (PBS) and incubated for one hour in Earle´s Balanced Salt Solution (EBSS, Sigma-Aldrich) for initial mTORC1 inactivation. Reactivation was initiated following a washing step by incubation in DMEM for one to two hours as indicated. Treatment with 250 nM Torin 1 (Cayman Chemical) during the reactivation period was used to silence mTOR kinase activity even in the presence of amino acids.

### Protein extraction and western blotting

Cultured cells were harvested by scraping into PBS supplemented with 5 mM EDTA and Complete protease inhibitor cocktail (Roche). After recovery by centrifugation, cells were lysed in 50 mM Tris-HCl, pH 7.4, 150 mM NaCl, 1% (w/v) Triton X-100, 0.1% (w/v) SDS, 4 mM EDTA supplemented with 4 mM Pefabloc (Roth), 1 µg/ml Pepstatin A (Sigma) and Complete (Roche). Samples were sonicated (level 4, 20 s) using a Branson Sonifier 450 (Emerson Industrial Automation) at 4°C and incubated on ice for 1 h. To remove insoluble material, samples were centrifuged (15000 g_max_, 10 min, 4°C) and total cell lysates recovered. Total organ lysates were obtained by homogenizing the tissues with an Ultra-Turrax in 50 mM Tris-HCl, pH 7.4, 150 mM NaCl, 4 mM EDTA supplemented with 4 mM Pefabloc, 1 µg/ml Pepstatin A, Complete (Roche) and PhosStop phosphatase inhibitor (Roche). After adjusting the samples to final concentrations of 1% (w/v) Triton X-100, 0.1% (w/v) SDS, they were sonicated as described above and kept on ice for 1 h. Finally, lysates were cleared by centrifugation (15000 g_max_, 15 min, 4°C) and lysates recovered. Protein concentration was determined with BCA (bicinchoninic acid) protein assay kit (Thermo Scientific). Electrophoretic separation of proteins by SDS-PAGE, the semidry transfer onto nitrocellulose and immunodetection were performed as described previously [8]. For the detection of TMEM192, membranes were blocked overnight at 4°C in 5% skim milk powder in TBS/T (20 mM Tris-HCl, pH 7.6, 150 mM NaCl, 1% (v/v) Tween-20) to reduce unspecific binding of the TMEM192-antibodies. Chemoluminescent signals were recorded with a LAS4000 imaging system (GE Healthcare) and quantified densitometrically with ImageJ. Western blots analyzing the mTORC1 target S6K were visualized and quantified using a LI-COR Odyssey^®^ Fc imager and the Image Studio™ software.

### Deglycosylation by PNGase F

To analyse potential N-glycosylation of TMEM192, 40 µg protein of total organ lysates (spleen, thymus, brain and liver) were denatured in PNGase puffer 1 (0.5% (w/v) SDS, 2% β-mercaptoethanol) for 5 min at 95°C. Subsequently, samples were adjusted to final concentrations of 50 mM sodium phosphate buffer, pH 8, 1.5% (v/v) Triton X-100, 0.2% (w/v) SDS, 10 mM EDTA, 0.8% (v/v) β-mercaptoethanol and, after adding 2 units of peptidyl N-glycosidase F (PNGase F, Roche) or 2 µl ddH_2_O, incubated at for 3 h 37°C. Finally, samples were subjected to Western blot analysis using anti-TMEM192 and anti-LIMP-2 as control.

### Determination of β-Hexosaminidase activity

Activity of b-Hexosaminidase was quantified spectrophotometrically in cell and tissue lysates as well as supernatants from cultured cells according to von Figura [28]. Samples were diluted in 0.9% (w/v) NaCl (final volume 25 µl) and mixed with an equivalent volume of substrate solution containing 10 mM p-Nitrophenyl-N-acetyl-β-D-glucosaminide (Sigma), 100 mM sodium citrate, pH 4.6, 0.2% (w/v) Triton X-100, 0.2% (w/v) BSA and 0.02% (w/v) NaN_3_. After incubation for 1 h at 37°C, the reaction was stopped by the addition of 200 µl 0.4 M Glycin-NaOH, pH 10.4. The absorption at 405 nm was measured in a Synergy HT microplate reader (BioTek Instruments Inc.) and used to calculate the corresponding enzyme activity. Based on the protein concentrations of the analysed lysates, the specific β-Hexosaminidase activities (mU/mg protein) were determined.

### Lysosomal exocytosis assay

Wild type and *TMEM192*-/- MEF cells were seeded in supplemented DMEM medium at a density of 200,000 cells per well of a 6-well plate. After adherence, the medium was replaced by phenol red-free DMEM (Gibco) devoid of serum. Cells were stimulated with 10 µM ionomycine for 10 min at 37°C or treated with an equivalent volume of DMSO as control. Immediately after the incubation, the supernatants were separated from the cells and centrifuged at 15,000 g_max_ for 5 min at 4°C to remove detached cells and cell debris. The cells were recovered by scraping and lysed with phenol red-free DMEM + 1% Triton X-100 and incubated on ice for 30 min. Afterwards, the cell lysates were cleared by centrifugation for 5 min at 15,000 g_max_ and 4°C. Activity of the lysosomal enzyme β-Hexosaminidase was determined in both media and cell lysates as described above.

### Indirect immunofluorescence

Immunocytochemical stainings of cultured cells were performed as described before [8]. In brief, cells adhered to coverslips were fixed with 4% (w/v) paraformaldehyde and subsequently permeabilised by including 0.2% saponin in all following incubations and washing solutions. Unspecific antibody binding was blocked by incubating the cells in 10% FCS diluted in PBS/0.2% Saponin, which was also used as diluent for primary and secondary antibodies. As primary antibodies rabbit anti-mTMEM192, rat anti-HA (3F10), mouse anti-hLAMP-2 (2D5), rabbit anti-mCathepsin D, rat anti mLAMP-2 (Abl93), mouse anti- LBPA (6C4) and rabbit anti LC3 were employed. These were visualized with the following fluorochrome-conjugated secondary antibodies: goat-anti-rabbit IgG Alexa 488 (mTMEM192, mCathepsin D, LC3), goat-anti-rat IgG Alexa 594 (HA, mLAMP-2) and goat-anti-mouse IgG Alexa 594 (hLAMP-2, LBPA). Nuclei were stained with DAPI (4-,6-diamidino-2-phenylindole, Sigma). To mount the coverslips Mowiol (Calbiochem) supplemented with the anti-fading reagents DABCO (1,4-diazobicyclo (2.2.2.) octane, Sigma) was used. Images of optical sections were acquired with an FV1000 confocal laser scanning microscope (Olympus).

### Histological analysis

Procedures for fixation and sectioning differed between different analyses. Details are given in the figure legends. In general, either mice were transcardially perfused with the respective fixative or native organs were fixed by immersion. Following perfusion, tissues were post-fixed overnight by immersion in the same fixative. As fixatives, either 4% (w/v) paraformaldehyde or 3% glutaraldehyde, both in 0.1 M phosphate buffer, pH 7.4, were employed. Specimens were either embedded into paraffin or araldite, as indicated, according to standard protocols. Staining of sections was performed with haematoxylin-eosin (HE) or toluidine blue. Alternatively, cryosections from fixed specimens were used for immunohistochemical visualization of β-galactosidase activity or immunohistochemical stainings. Therefore, prior to freezing and sectioning fixed tissues were incubated overnight in 30% (w/v) sucrose in PBS at 4°C. Cryosections of 30 µm thickness were subjected to the staining procedures. X-Gal staining of β-galactosidase activity was carried out by permeabilizing the brain sections for 10 min in 0.01% Na-Deoxycholat, 0.02% NP40 in PBS. After washing for 10 min in PBS, the sections were incubated in X-Gal-solution (5 mM K_3_Fe(CN)_6_, 5 mM K_4_Fe(CN)_6_, 2 mM MgCl_2_, 1 mg/ml X-Gal in PBS) for 2.5 h at 37°C. For immunohistochemical detection of TMEM192 in brain cryosections, endogenous peroxidase activity was quenched by incubation for 30 min in 1.6% H_2_O_2_ in TBS. After permeabilization by washing four times for 10 min with washing buffer (0.25% (w/v) Triton X-100 in PBS), unspecific binding was blocked by 4% Normal Goat Serum, 0.5% (w/v) Triton X-100 in PBS for 1 h. The primary antibody (anti-TMEM192) was applied overnight at 4°C. The next day, the brain sections were washed four times for 10 min in washing buffer and then incubated for 1 h in biotinylated secondary antibody. DAB-staining was performed using DAB Peroxidase Substrate Kit (Vector Labs). After washing the sections five times in washing buffer for 10 min the sections were incubated in ABC solution for 1 h. Before adding DAB-solution brain sections were washed four times in PBS. In order to stop the DAB-reaction PBS was added after 5-10 min. The stained brain sections were photographed with a Leica DMi8 microscope.

### Electron microscopy

Livers from mice transcardially perfused with 3% glutaraldehyde in 0.1 M phosphate buffer, pH 7.4, were postfixed in the same solution. Tissue was additionally postfixed with 2% OsO_4_ and embedded in araldite according to standard protocols. Ultrathin sections were stained with uranyl acetate and lead citrate.

### Flow cytometric analysis

To obtain single-cell suspensions, murine spleen, thymus, lymph nodes and red bone marrow isolated from femur and tibia were passed through a 100 µm cell strainer (BD Biosciences) and suspended in ice-cold MACS buffer (0.5% BSA, 2 mM EDTA in PBS). Next, suspensions of spleen, thymus and bone marrow were incubated in erythrocyte lysis puffer (150mM NH_4_Cl, 15 mM Na_2_CO_3_, 0.1 mM EDTA, pH 7.3) for 12 min at room temperature. Cells were stained for 30 min at 4°C with the following FITC-, PE- or APC-conjugated murine antibodies diluted in MACS buffer: anti-B220 (RA3-6B2), anti-CD3e (145-2C11), anti-CD11c (N418), anti-CD21 (eBio4E3), anti-CD43 (eBioR2/60), anti-F4/80 (BM8), anti-IgM (II/41), anti-MHCII (M5/114.15.2) (all from eBioscience); anti-B220 (S4401), anti-CD4 (220 126), anti-CD8a (150 898), anti-Gr1 (220 220), anti-NK-cells (S4241) (all from ImmunoTools); anti-CD11b (M1/70), anti-IgD (11-26c.2a) (all from BD); anti-CD23 (B3B4), anti-CD24 (M1/69) (all from BioLegend). Stained cells were washed in 0.5 µg/ml propidium iodide (BD) diluted in MACS buffer to label dead cells (PI^+^) and analysed using a FACS Canto or FACS Canto II flow cytometer (BD). Data were analysed with FACS Diva (BD) or FlowJo (Three Star) software.

## SUPPLEMENTARY MATERIALS FIGURES


