# Supplementary material for: Functional characterization of the lysosomal membrane protein TMEM192 in mice
Source: Oncotarget. 2017 Apr 28;8(27):43635–52. doi: 10.18632/oncotarget.17514 (PMC5546430; doi:10.18632/oncotarget.17514)
Supplement: Supplementary file 1 [file oncotarget-08-43635-s001.pdf]

Functional characterization of the lysosomal membrane protein TMEM192 in mice

Supplementary Material

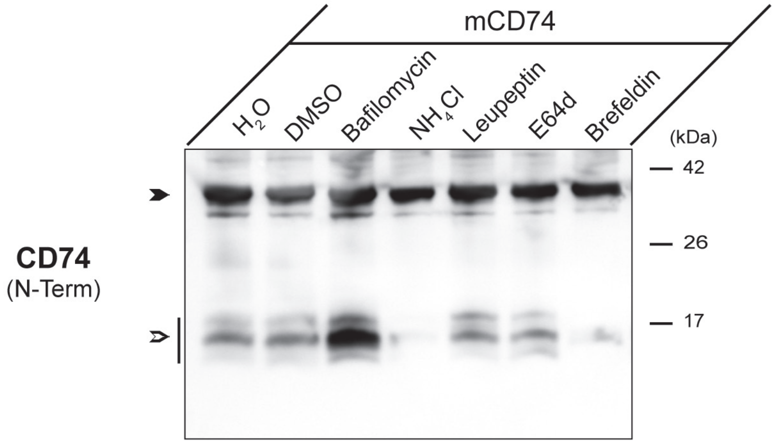

Supplementary Figure 1: Effect of the applied inhibitors on processing of the invariant chain CD74. HeLa cells were transiently transfected with the p31 isoform of murine CD74. As indicated, cells were treated overnight with Bafilomycin (30nM), NH_4Cl (25 mM), Leupeptin (100 µM), E64d (40 µM) or Brefeldin A (1 µg/ml) and H_2O or DMSO as negative control. Concentrations of the inhibitors and experimental conditions were equivalent to the analysis of TMEM192 processing depicted in Figure 3B. Aliquots of total cell lysates were analysed by Western blotting using an antibody directed against an N-terminal epitope of CD74 located within its cytoplasmic domain. Consequently, this antibody detected the CD74 full-length protein (closed arrowhead) as well as different N-terminal fragments (NTFs, open arrowhead). Whereas Brefeldin A and NH_4Cl abolished the generation of any NTFs, Bafilomycin induced a significant NTF stabilisation. In contrast, effects of E64d and Leupeptin were more subtle leading to an increased abundance of the upper NTF band. A variety of endosomal/lysosomal proteases, which differ in their pH dependence, have been implicated in the processing of CD74 which accounts for these effects. Importantly, these observations confirm the effectiveness of the applied compounds in the experimental system utilised for the analysis of TMEM192 proteolysis a depicted in Figure 3B.

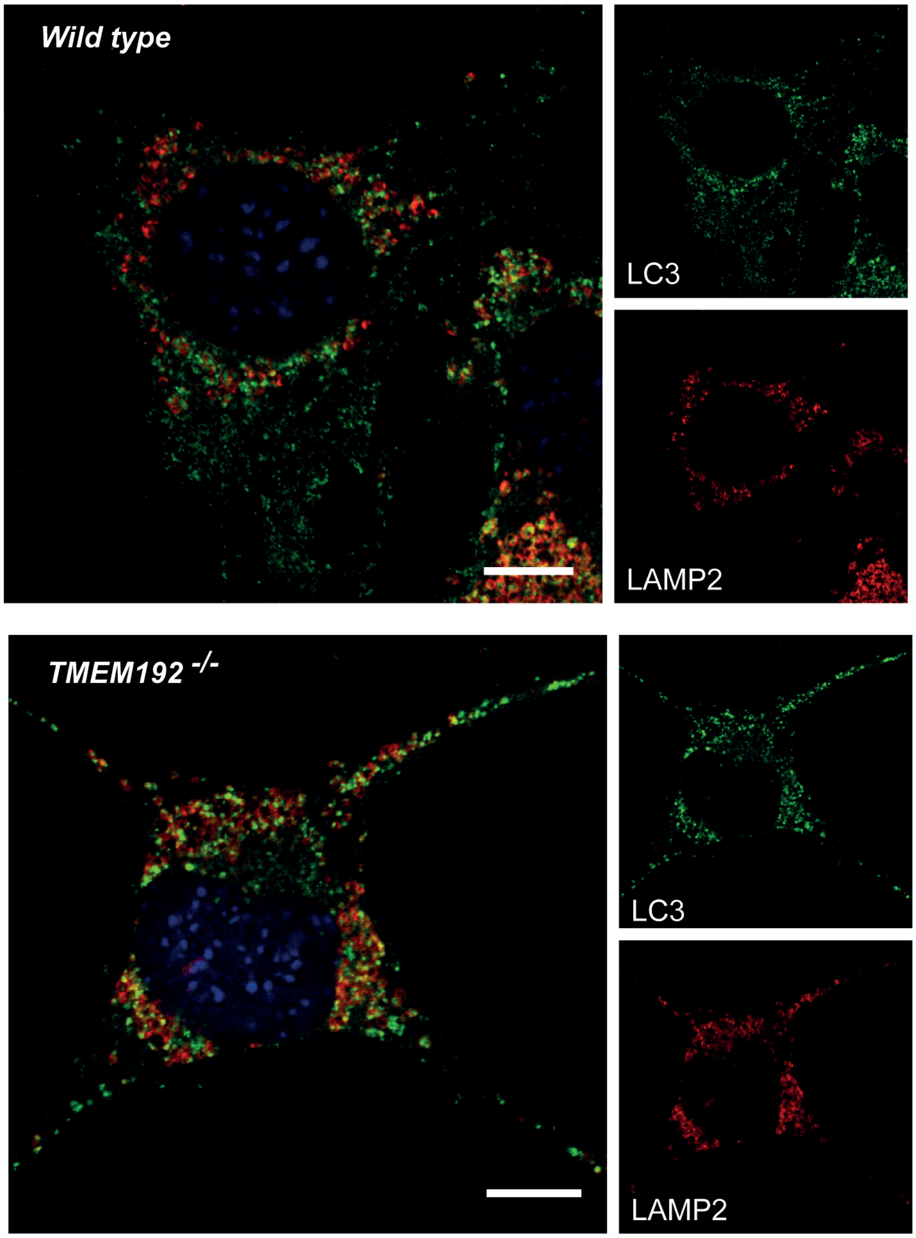

Supplementary Figure 2: Autophagic cargo can be delivered to lysosomes in TMEM192-deficient murine embryonic fibroblasts (MEFs). Wild type and *TMEM192*$^{-/-}$ MEFs were starved for 3 h in EBSS in the presence of 30 nM Bafilomycine. After fixation, LC3 and LAMP2 were visualized by indirect immunofluorescence. Nuclei were stained with DAPI. In cells of both genotypes a partial co-localization of LC3 and LAMP2 was observed. Scale bar, 10 µm.

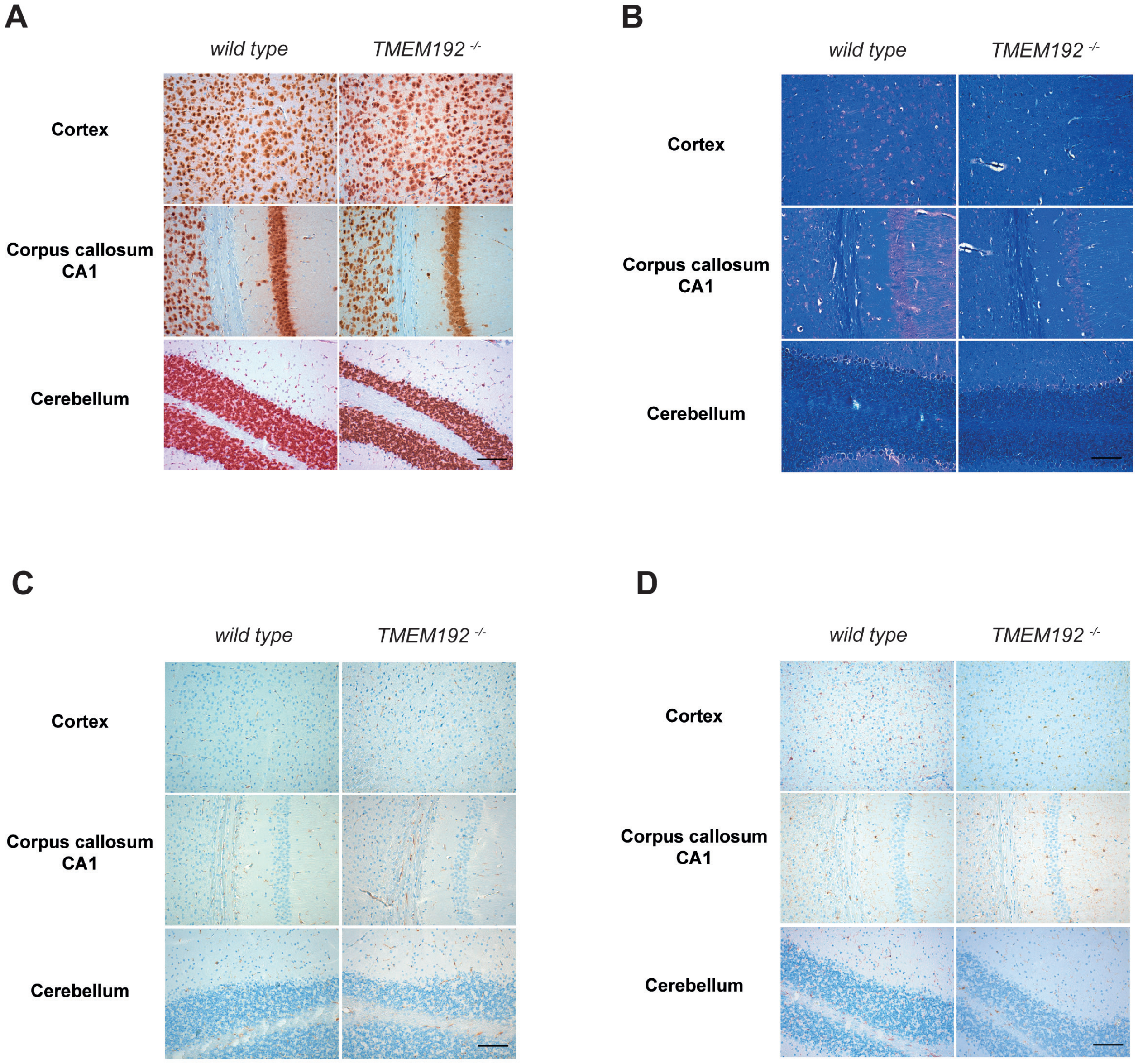

Supplementary Figure 3: Histological analysis of brains from wild type and TMEM192-/- mice. Representative paraffin sections of cortex, cerebellum and hippocampus from immersion-fixed brains (4% PFA) were analysed. Immunohistochemical stainings against the neuronal marker protein NeuN (A), the astrocyte specific protein GFAP (glial fibrillary acidic protein) (B) and microglia-specific protein Iba1 (C) were performed. These demonstrated regular neuronal distribution in the *TMEM192*$^{-/-}$ brains as well as absence of astrocytosis and microglial activation. Furthermore, myelination as determined by luxol blue staining (D) was comparable in wild type and *TMEM192*$^{-/-}$ brains. Scale bar: 100 µm
